# Decline in plankton diversity and carbon flux with reduced sea ice extent along the Western Antarctic Peninsula

**DOI:** 10.1038/s41467-021-25235-w

**Published:** 2021-08-16

**Authors:** Yajuan Lin, Carly Moreno, Adrian Marchetti, Hugh Ducklow, Oscar Schofield, Erwan Delage, Michael Meredith, Zuchuan Li, Damien Eveillard, Samuel Chaffron, Nicolas Cassar

**Affiliations:** 1 Division of Earth and Ocean Sciences, Nicholas School of the Environment, Duke University, Durham, NC USA; 2 Université de Brest—UMR 6539 CNRS/UBO/IRD/Ifremer, Laboratoire des sciences de l’environnement marin—IUEM, Rue Dumont D’Urville, Plouzané, France; 3 Environmental Research Center, Duke Kunshan University, Kunshan, China; 4 Department of Marine Sciences, University of North Carolina at Chapel Hill, Chapel Hill, NC USA; 5 Lamont-Doherty Earth Observatory, Columbia University, Palisades, NY USA; 6 Rutgers University’s Center for Ocean Observing Leadership (RU COOL), Department of Marine and Coastal Sciences, School of Environmental and Biological Sciences, Rutgers University, New Brunswick, NJ USA; 7 Université de Nantes, CNRS UMR 6004, LS2N, Nantes, France; 8 British Antarctic Survey, Cambridge, United Kingdom; 9 Applied Ocean Physics and Engineering, Woods Hole Oceanographic Institution, Woods Hole, MA USA; 10 Research Federation for the Study of Global Ocean Systems Ecology and Evolution, FR2022/Tara Oceans GOSEE, Paris, France

**Keywords:** Water microbiology, Carbon cycle, Marine biology

## Abstract

Since the middle of the past century, the Western Antarctic Peninsula has warmed rapidly with a significant loss of sea ice but the impacts on plankton biodiversity and carbon cycling remain an open question. Here, using a 5-year dataset of eukaryotic plankton DNA metabarcoding, we assess changes in biodiversity and net community production in this region. Our results show that sea-ice extent is a dominant factor influencing eukaryotic plankton community composition, biodiversity, and net community production. Species richness and evenness decline with an increase in sea surface temperature (SST). In regions with low SST and shallow mixed layers, the community was dominated by a diverse assemblage of diatoms and dinoflagellates. Conversely, less diverse plankton assemblages were observed in waters with higher SST and/or deep mixed layers when sea ice extent was lower. A genetic programming machine-learning model explained up to 80% of the net community production variability at the Western Antarctic Peninsula. Among the biological explanatory variables, the sea-ice environment associated plankton assemblage is the best predictor of net community production. We conclude that eukaryotic plankton diversity and carbon cycling at the Western Antarctic Peninsula are strongly linked to sea-ice conditions.

## Abstract (web summary)

Over the past century, the Western Antarctic Peninsula has experienced rapid warming and a substantial loss of sea ice with important implications for plankton biodiversity and carbon cycling. Using a 5-year DNA metabarcoding dataset, this study assesses how interannual variability in sea-ice conditions impacts biodiversity and biological carbon fluxes in this region.

## Introduction

**Fig. 1:**
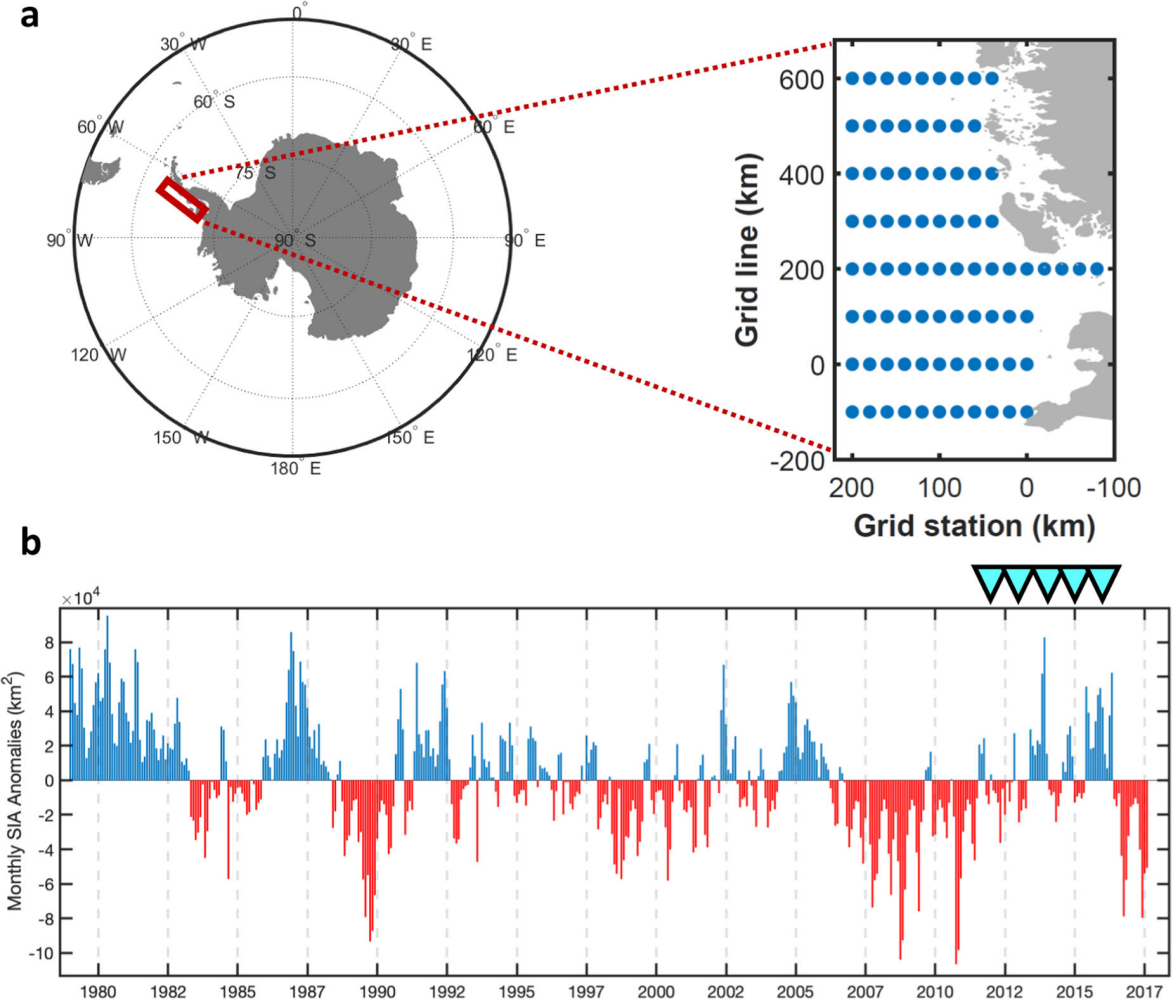
Monthly sea-ice area anomalies at the WAP. **a** Location of the study area (red box) and the Palmer LTER sampling grid with hydrostations (blue dots). **b** Time-series of monthly averaged sea-ice area (SIA) anomalies for Palmer LTER sampling area from 1979 to 2017. Blue (red) bars represent negative (positive) SIA compared to a 39-year climatology for a particular month. SIA data were downloaded from Palmer LTER DataZoo (http://pal.lternet.edu/data). Cyan arrows highlight the sampling periods in this study.

The Southern Ocean disproportionally contributes to the global climate system, accounting for almost half of the anthropogenic CO_2_ and 75% of the heat uptake by the oceans^1,2^. The Western Antarctic Peninsula (WAP) system has exhibited some of the most significant changes in the Southern Ocean^3,4^, with rising air temperature up to 7 °C since 1950^5^, warming and freshening of the upper ocean^6^, warming of the deeper ocean^6^, deepening of the mixed layer depth (MLD)^7^, and the fastest sea ice decrease in Antarctica (Fig. 1)^8,9^. Whilst atmospheric warming trends at the Antarctic Peninsula have paused or even reversed in places since the end of the twentieth century, this is understood as natural interannual climate variability that is superposed on the longer-term trends^10^. There have been observed ecosystem changes throughout the entire Antarctic marine food web^7,11–14^. At the base of the food web, WAP eukaryotic plankton including phytoplankton and microzooplankton support higher trophic levels ranging from krill to penguins and whales^13^, drive biogeochemical cycles^15–17^, and regulate oceanic carbon uptake^7^. Thus, given the fundamental importance of eukaryotic plankton at the WAP, it is imperative to understand and predict the changes in plankton community structure, biodiversity, and carbon flux in this rapidly changing environment^18^.

The WAP system is characterized by a short but highly productive growing season during austral spring and summer^19^. The net community production (NCP) represents the balance between gross primary production and community respiration. When the organic carbon pool at the mixed layer is under a steady state, the net carbon flux in (i.e., NCP) equals the net carbon flux out (i.e., carbon export). Therefore, NCP reflects the amount of organic carbon available for export out of the surface MLD.

Here, we analyze five years of high-resolution NCP and high-throughput DNA sequencing data to explore the contribution of polar eukaryotic plankton to biological carbon fluxes. We show that among the considered environmental factors (iron not included), SST and sea-ice condition are strong predictors for community structure and NCP. We find that biodiversity is reduced when SST is high at the WAP. Finally, in order to improve NCP predictions, we build machine-learning models including in-depth community structure, community co-occurrence patterns, and physical conditions. Among the top-performing NCP models, a sea-ice associated plankton assemblage is a key predictor, with central (i.e., most connected) taxa identified as *Thalassiosira*, *Odontella*, *Porosira*, *Actinocyclus*, *Proboscia*, *Chaetoceros,* and *Gyrodinium*. The combination of biogeochemical tools and DNA metabarcoding sheds a unique insight into environmental forcings, plankton diversity, community structure and interaction, and biological carbon flux variability in a rapidly changing polar environment.

## Results and discussion

**Fig. 2:**
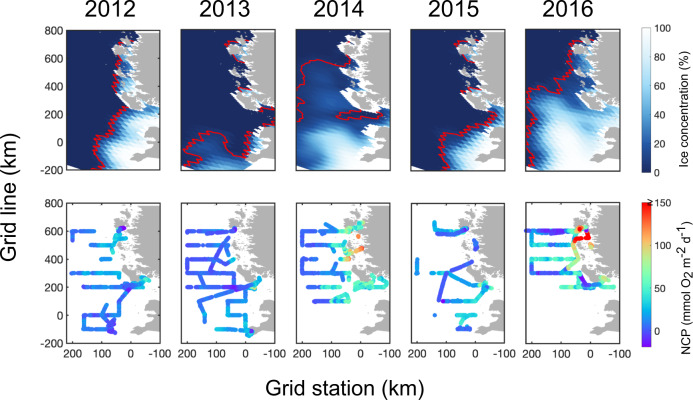
Spatial patterns of summer WAP ice concentrations and NCP from 2012 to 2016. January averaged sea-ice concentrations derived from passive microwave satellite measurements (*top*). Red contours represent the biologically relevant ‘ice-edge’ defined as an ice concentration threshold of 5%. Underway estimates of NCP using O_2_/Ar method from the annual PAL-LTER sampling cruises along the WAP grids (*bottom*).

O_2_/Ar-based in situ NCP observations at the WAP in austral summer from 2012 to 2016 demonstrate substantial spatial heterogeneity and interannual variability (Fig. 2). This was a period of moderately positive sea-ice area (SIA) anomalies following a more prolonged period of anomalously low SIA (Fig. 1). During the summer, NCP was highest in the shelf zone and decreased offshore, which is consistent with previous ship-based^15,20^ and satellite-based observations^19^. In addition, the observed NCP exhibited marked interannual variability related to ice conditions. The two years that feature late sea-ice retreat (2014 and 2016) were associated with abnormally high summer NCP (t-test, *p* < 0.0001) (Fig. 2). In previous studies, elevated NCP or primary production under high sea-ice conditions were attributed to ice-melt enhanced water column stability, thus higher light availability, and potential iron supplied by sea ice^7,20^. From a decadal study considering climate oscillations^13^, bloom-favorable conditions at the WAP have been linked to negative winter and spring phases of the Southern Annular Mode (SAM), the dominant mode of extra-tropical climate variability in the Southern Hemisphere^21^. Negative SAM leads to increased ice extent in winter, restricting deep mixing, and then enhanced ice-melt in spring/summer, resulting in intensified stratification.

Eukaryotic plankton, including phytoplankton and microzooplankton, are key drivers of carbon fluxes at the WAP^16^. Based on a five-year WAP DNA sampling, we explored the plankton community structure and diversity via sequencing of the 18S rRNA gene marker. At the phylum level, four eukaryotic plankton dominated the WAP surface water, including diatoms (25.0%), cryptophytes (23.0%), dinoflagellates (19.6%), and haptophytes (11.3%) (Fig. S3). Other eukaryotic plankton groups, mostly heterotrophic protists, contributed less than 5% of the 18S reads. Community composition differed substantially between years with high and low sea ice extent (Fig. S4). For the years 2014 and 2016 with high sea ice, eukaryotic plankton communities comprised on average 39.5 ± 3.0% diatoms, 20.5 ± 1.3% dinoflagellates, 15.1 ± 6.1% cryptophytes, and 7.1 ± 2.4% haptophytes. In contrast, for warm years with less sea ice in 2012, 2013, and 2015, eukaryotic plankton communities comprised on average 28.9 ± 8.3% cryptophytes, 19.0 ± 1.6% dinoflagellates, 14.4 ± 4.4% haptophytes, and 14.0 ± 1.7% diatoms, all significantly different from cold years (two-sided t-test, *p* < 0.0001).

**Fig. 3:**
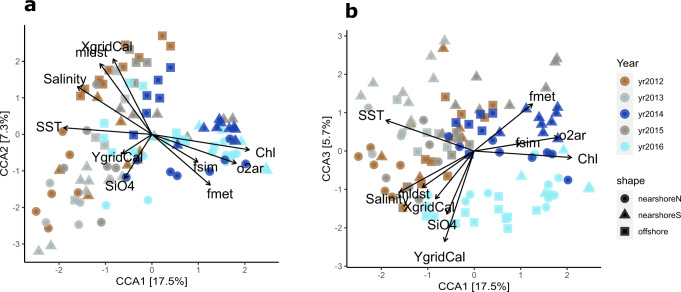
Canonical correspondence analysis (CCA) biplots. Each point represents the eukaryotic plankton community composition from a surface ocean sample with year indicated by color and station type (i.e., offshore, nearshore north, or nearshore south) indicated by shape. CCA1 and 2 are depicted (**a**) as well as CCA1 and 3 (**b**). Vectors indicate stepwise selected environmental constrains, both biotic and abiotic, with factor names marked at the end. Acronyms for selected environmental factors: SST – sea surface temperature, SiO4 – silicate concentration, mldst – mixed layer depth defined by potential density, XgridCal – X grid or grid station calculated from GPS, YgridCal – Y grid or grid line calculated from GPS, *f*_sim_ – fraction of sea-ice melt estimated from δ^18^O, *f*_met_ – fraction of meteoric water estimated from δ^18^O, Chl – chlorophyll concentration, o2ar – biological oxygen supersaturation. Source data are provided as a Source Data file.

At the finest taxonomic resolution, 2480 amplicon sequence variants (ASVs) were identified from the five-year amplicon dataset (119 samples). Canonical correspondence analysis (CCA) illustrates that either ice conditions or SST is the dominant driver on community structure at the ASV level (Fig. 3). The first axis CCA1 (17.5% of the variance) separates samples from late (2014 and 2016) and early (2012, 2013, and 2015) ice-retreat years. The most substantial abiotic factor associated with CCA1 is SST (negatively correlated), and the most substantial biotic factors associated with CCA1 are Chl and biological O_2_ (both positively correlated), consistent with ice-melt enhanced biomass and productivity. Freshwater inputs were estimated from oxygen isotope signatures. Low salinity, as well as high fractions of sea-ice melt and meteoric water, are also associated with CCA1 but to a lesser extent than SST. CCA2 (7.3% of the variance) separates mainly the offshore and nearshore samples, with distance to coast (X grid) and MLD being the top two associated environmental factors. CCA3 (5.7% of the variance) indicates community differentiation along the north-to-south gradient (Y grid), potentially reflecting a long-term ice retreat impact on communities and/or a north-to-south climate gradient along the WAP^11,19^; CCA3 could also reflect interannual variability in sea ice extent. Overall, sea-ice conditions and associated environmental parameters, such as low SST (Fig. S1 and S2) and low salinity, are the primary drivers of community differentiation at the ASV level.

**Fig. 4:**
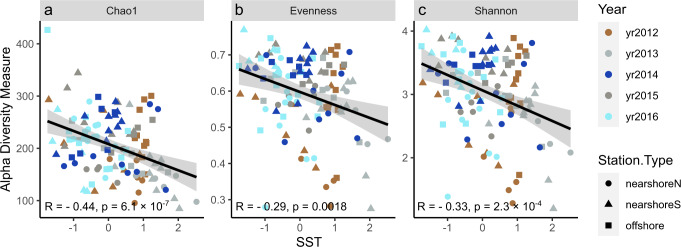
Alpha diversity indices vs. SST. Three diversity indices, including Chao1 (**a**), Pielou’s evenness (**b**), and Shannon (**c**), plotted against SST, with year indicated by color and station type indicated by shape. Reads from each sample were rarefied to an even depth. Solid red lines represent linear fittings and the gray bands represent 95% confidence intervals. All three diversity indices show significant negative correlations with SST (two-sided t-test, *p* < 0.01). Source data are provided as a Source Data file.

To further investigate the effect of temperature as one of the major abiotic factors influencing polar plankton composition^22^, we examined the temperature effect on biodiversity (ASV based) using three diversity indices, Chao1, Pielou’s evenness, and Shannon. Chao1, a measure for species richness, demonstrated an evident decline towards higher SST (Fig. 4), with a 40% decrease in the index for a 4 °C rise in SST. Pielou’s evenness and Shannon, which consider both species richness and evenness, also decreased significantly with increasing SST. It indicates that communities in warmer WAP waters show lower richness and lower evenness, i.e., that a few taxa dominate. Interestingly, in a recent global analysis on plankton biodiversity from *Tara* Oceans^23^, the temperature was also identified as the major explanatory factor for global-scale eukaryotic plankton biodiversity estimated by the Shannon index, but with the opposite trend, i.e., a decreased diversity towards higher latitude or lower temperature. We note that whilst the *Tara* Oceans dataset represents the most comprehensive oceanic DNA sampling efforts to date, it featured only limited sampling in the Southern Ocean (three data points), and it did not include a longitudinal survey that captures the mesoscale effect of changing temperature on a given community; our five-year WAP sample collection hence complements well the *Tara* Oceans observations for the previously under-sampled Southern Ocean. One explanation for the unexpected high biodiversity observed under low temperature at the WAP is that the ice-associated plankton communities consist mainly of diatoms (Fig. S4), which are highly diverse and can thrive at lower temperatures compared to other phytoplankton^24^. As a unique longitudinal test case (e.g., as documented in^25^), our results suggest that global warming may decrease plankton diversity in coastal Antarctica.

Due to the high dimensionality of the ASV dataset, it is challenging to model NCP based on community structure. Thus, we applied a weighted gene correlation network analysis (WGCNA) approach to delineate clusters of 18S ASVs into subnetworks or modules (Fig. S5)^26,27^. This approach allows us to reduce the total number of variables while preserving information on ASV abundances and potential interactions^28^. In total, we identified 12 modules from the five-year global community structure (Fig. S5). Each module represents an assemblage of predicted highly interconnected plankton community members, potentially indicating a group of organisms with strong ecological overlap and/or interactions^29^. The eigenvalue of each module represents the overall abundance of the assemblage. In addition, in order to investigate the niche partitioning of the different community assemblages or modules, correlation analysis was performed in WGCNA to link them to different abiotic and biotic factors (Fig. S5). Next, we applied Genetic Programming (GP), a machine learning approach based on evolution computation^30,31^, to generate and parameterize statistical models that predict NCP based on the WGCNA generated bio-assemblages (*n* = 12) and physical factors (*n* = 6) (see “Methods” for a detailed list). The relationships between carbon-based plankton biomass, their physiology (indirectly modeled as functions of environmental factors), and biogeochemical rates are often non-linear and could involve multiple layers of interactions. GP allows us to capture the complex and non-linear relationships between these different factors to predict NCP without an a priori assumption. The overall idea of this modeling approach is that biogeochemical rates are a function of (i) the community composition and abundance; and (ii) the specific metabolic rates regulated by environmental factors, such as the photosynthesis-irradiance curve and the productivity−temperature (*Q*_10_) relationship. The top four GP solutions (Table S2, ranked by mean square error (MSE)) provide good predictions on NCP with *R*^2^ ranging from 0.70 to 0.80. Among the explanatory variables selected by the models, the top two physical factors ranked by selection frequency are SST and MLD, followed by surface photosynthetically active radiation (PAR). This suggests that temperature and light are likely the primary physiological limiting factors on NCP in the WAP system. As an alternative, SST could be an indirect proxy for time since ice-retreat, i.e., higher SST indicates a longer time after the initial ice retreat.

**Fig. 5:**
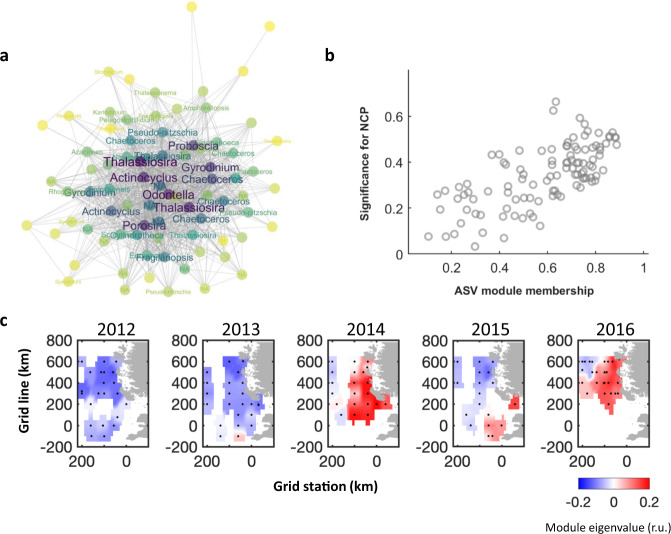
An ice-associated plankton assemblage (MET module from WGCNA analysis). **a** The MET subnetwork presents a diverse group of correlated ASVs or nodes, with each node colored by its centrality (i.e., darker color for higher centrality). The hub nodes of the network, i.e., the top-10 ASVs with the highest connectivity or the central members for the network, were identified at the genus level as *Thalassiosira*, *Odontella*, *Porosira*, *Actinocyclus*, *Proboscia*, *Chaetoceros,* and *Gyrodinium*. **b** ASVs with higher module membership, i.e., higher intramodular connectivity, are more correlated with volumetric NCP (*R* = 0.7, two-sided t-test *p* = 9.6 × 10^−16^). **c** The biogeography of MET at the WAP, with color indicating the eigenvalue in the relative unit. Black points show the DNA sampling locations. Source data are provided in Supplementary Data 2 and 4.

Among the community-assemblage factors in GP solutions, module turquoise (MET) is the most important predictor for NCP. MET is also the largest module identified, which consists of 126 ASVs, mainly representing diverse groups of diatoms and dinoflagellates (Fig. 5a; Supplementary Data 2). The central nodes in the MET network, which represent the top-10 most connected ASVs or the central ASVs for the network structure^32^, include the diatom genera *Thalassiosira*, *Odontella*, *Porosira*, *Actinocyclus*, *Proboscia*, *Chaetoceros*, and the dinoflagellate *Gyrodinium*. MET appears in all top-four performed GP solutions, and it is the sole biological factor in solution 1 explaining a majority of the NCP variability (*R*^2^ = 0.70) (Table S2). The overall spatial distributions of MET are consistent with the January averaged sea-ice distribution estimated by satellite (Fig. 5c). In the WGCNA correlation analysis (Fig. S5), MET is significantly correlated with low SST (*R* = −0.51, *p* = 1 × 10^−8^), low salinity (*R* = −0.48, *p* = 8 × 10^−8^), shallow MLD (*R* = −0.36, *p* = 1 × 10^−4^), elevated sea-ice melt (*R* = 0.33, *p* = 3 × 10^−4^) and meteoric freshwater (*R* = 0.34, *p* = 3 × 10^−4^). Although sea-ice melt and meteoric freshwater both show positive effects on MET, they could exert this through different mechanisms. Because iron concentrations were not included in this analysis, we cannot discern whether sea ice and/or glacial ice melt impact productivity through altering light and/or iron levels by increased stratification or fertilization. However, according to a recent study at the eastern Antarctic Peninsula, sea-ice melt could mostly influence carbon fixation through water column stabilization, while the effect of glacial melt could be through providing a significant amount of iron to the system^33^. In the Southern Ocean, photosynthetic efficiency (Fv/Fm) varies with an iron status where lower values suggest iron stress and higher values of iron sufficiency^34^. Previous studies have found NCP to be positively correlated with Fv/Fm^35^. Although no direct iron measurements were made in this study, iron availability being a first-order factor regulating NCP at the WAP cannot be ruled out. Furthermore, besides NCP (*R* = 0.57, *p* = 8 × 10^−11^) ship-based observations of primary production (PP) and bacterial production (BP) are also both positively correlated with MET with *R* = 0.47, *p* = 1 × 10^−7^ and *R* = 0.61, *p* = 2 × 10^−12^, respectively. This indicates that MET-dominated regions have high biological activities. The high NCP values associated with MET are largely driven by autotrophs; otherwise, we would expect a negative correlation between MET/NCP and BP.

Two other community-assemblages, MER (module red, 27 ASVs) and MEG (module green, 29 ASVs), also contribute to NCP models but to a lesser extent (Table S2). In the GP solutions (1) and (2), MER contributes to NCP positively, and the addition of MER marginally improves NCP prediction (*R*^2^ from 0.70 to 0.72, MSE from 1.18 to 1.12). The MER assemblage is dominated by the cryptophyte *Geminigera* that appear in warmer waters (SST, *R* = 0.45, *p* = 6 × 10^−7^) and towards the north WAP (Y grid, *R* = 0.4, *p* = 2 × 10^−5^) (Figs. S5 and S6). MEG contributes to NCP negatively in GP solutions (2) and (3). It represents a group of heterotrophic protists, dominated by *Picomonas* and *Telonema*. The top two environmental factors correlated with MEG are MLD (*R* = 0.5, *p* = 2 × 10^−8^) and distance to shore (X grid, *R* = 0.49, *p* = 6 × 10^−8^).

Based on our observations and analyses, we hypothesize that the summer plankton community—NCP system at the WAP mainly follows three broad patterns: (i) large centric diatoms associated with ice-melt form intensive blooms and fuel a short food chain from krill to other top predators^13^. In particular, the spring melt of sea ice and glacial discharge could work in concert to stabilize the water column and provide a source of iron. This high productivity combined with small losses through trophic transfer results in high export production. (ii) In warmer water, small cryptophytes dominate. Compared to large diatoms, their growth could be more efficiently checked by small microzooplankton grazers^36^, thus resulting in lower biomass for export^16^. Moreover, the food chain starting from small phytoplankton is longer due to more trophic level transfers, and the organic matter could be more subject to remineralization^37^. (iii) With deep mixing, primary production in the water column is low due to light limitation. Because of the limited food resource, heterotrophic protists feeding on bacteria and detritus dominate the microzooplankton system. Compared to scenarios (i) and (ii), more organic carbon may be recycled through the microbial loop, which further reduces carbon export and air−sea CO_2_ fluxes^7^. The last pattern displays the lowest NCP. Previous WAP studies using an inverse food web model illustrated that microzooplankton grazing and the microbial loop could consume a significant amount of carbon^38,39^. With climate change, the WAP region is projected to have a significant loss in summer sea ice, a rise in sea surface temperatures, and deeper mixing associated with more open water and stronger winds. Consequently, the latter two scenarios may become more prevalent in the upcoming years to decades.

Although our study represents the longest record of eukaryotic DNA-based community structure and NCP in coastal Antarctica, our observations are limited to seasonal snapshots of the (summer) WAP system. These observations need to be expanded to larger spatial and temporal scales in the Southern Ocean. In the future, correlation-based analyses and statistical models will need to be further validated with field incubations and lab experiments. Non-targeted omics-based surveys (e.g., metagenomic, metatranscriptomic, and proteomic studies) will provide additional insights into the microbial metabolic pathways, which are directly linked to the biogeochemical rates and associated ecosystem functions. Moreover, they need to be coupled with high-resolution time-series studies to help us unravel changes in phytoplankton phenology and predator-prey dynamics. Despite the methodological limitations and uncertainties, our results indicate that temperature and sea ice extent are two important environmental factors regulating the summer WAP eukaryotic plankton community structure, biodiversity, productivity, and associated carbon export potential. To the extent that the observed interannual variability in the influence of sea ice extent on ecosystem structure and functioning serves as a proxy for broader, longer-term ecological consequences associated with climate change, the WAP and other coastal Antarctica regions could be destined for reduced biodiversity and biological carbon drawdown. However, a longer time series will be needed to confirm the pattern.

## Methods

### Environmental data and DNA sampling

Environmental data from the Palmer Long-Term Ecological Research (LTER) cruises can be accessed from the online data repository Palmer LTER DataZoo (http://pal.lternet.edu/data). The detailed sampling methods and in situ biological rates measurements were described previously in^40^. In brief, each year in January, a research vessel conducted intensive oceanographic and biological surveys across the shelf-transects and a north−south gradient at the West Antarctic Peninsula (WAP). During the annual LTER cruises, underway measurements and surface water sampling were conducted from the ship’s continuous flow-through system. Discrete water samples in-depth profiles were collected using a Conductivity−Temperature−Depth (CTD) rosette. Mixed layer depth (MLD) was estimated from the ship’s CTD profiles by Δσ_θ_ = 0.03 kg m^−3^ using a threshold method^41^.

PAR above the water was continuously recorded from the mast PAR sensor of the ship. It was converted to PAR just beneath the water surface using a constant of 0.92. Average PAR in the mixed layer (PAR_mld) was then calculated following the method described in^42^.

Freshwater fractions were estimated from salinity and oxygen isotope signatures (δ^18^O) in seawater detailed in^43^. In brief, sampled seawater was assumed to be a mixture of ice-melt, meteoric meltwater, and Circumpolar Deep Water (CDW). A three-end member mass balance method was used to calculate the fractions, with salinity and δ^18^O values in 7 and 2.1‰ for sea-ice melt, 0 and −16‰ for meteoric meltwater, and 34.73 and 0.1‰ for CDW.

In order to collect eukaryotic plankton DNA, surface seawater from the ship’s underway flow-through system was gently vacuum-filtered onto a 47 mm, 0.45 µm Supor filter (Pall Corporation, New York, NY, USA) for years 2012 and 2013, or a 47 mm, 0.2 µm Supor filter for years 2014, 2015, and 2016. The filtration volumes were about 4 L or less at high biomass stations. For each filtration, the exact filtrate volume was recorded for later quantitative microbiome profiling (QMP). The filters were immediately stored at −80 °C until further analysis.

### Remote sensing data

January sea ice concentrations from 1979 to 2020 were downloaded from the National Snow and Ice Data Center website https://nsidc.org/. The data are in the polar stereographic projection, with each grid representing a 25 × 25 km area. January sea surface temperature (SST) data from 1982 to 2012 were acquired by the AVHRR and downloaded from NOAA website https://www.ncei.noaa.gov/. January SST data from 2013 to 2020 were acquired by MODIS-Aqua and downloaded from NASA ocean color website https://oceancolor.gsfc.nasa.gov/. SST data have a spatial resolution of 4 × 4 km in the equatorial region. Finally, we extracted January SST and sea ice concentrations in the Palmer grid from lines 0 to 900 and stations 0 to 220 (Figs. S1 and S2).

### Underway O_2_/Ar—NCP measurements

The O_2_ concentration in the mixed layer is influenced by physical and biological processes. Using Ar, an inert gas with similar solubility properties as O_2_, we decomposed total O_2_ into physical and biological components. Seawater O_2_/Ar ratios were measured underway from the ship’s flow-through system, using an equilibrator inlet mass spectrometer (EIMS)^44^. Biological O_2_ supersaturation was estimated as

$$\Delta ({{{{{{\mathrm{O}}}}}}}_{2}/{{{{{\mathrm{Ar}}}}}})=\left[\frac{({{{{{{\mathrm{O}}}}}}}_{2}/{{{{{\mathrm{Ar}}}}}}){{{{{\mathrm{sample}}}}}}}{({{{{{{\mathrm{O}}}}}}}_{2}/{{{{{\mathrm{Ar}}}}}}){{{{{\mathrm{sat}}}}}}}-1\right]\times 100 \%$$

High-resolution NCP in units of mmol O_2_ m^−2^ day^−1^, was then derived from Δ(O_2_/Ar) and NCEP reanalysis winds as previously described in^45^, except for a modification to the gas exchange weighting following^46^. NCP estimation can be expressed as the equation below,

$${{{{{\mathrm{NCP}}}}}}=k\left[{{{{{{\mathrm{O}}}}}}}_{2}\right]{{{{{\mathrm{sat}}}}}}\Delta ({{{{{{\mathrm{O}}}}}}}_{2}/{{{{{\mathrm{Ar}}}}}})$$

Where *k* denotes the gas transfer velocity for O_2_ (estimated based on^47^) and [O_2_]_sat_ is the equilibrium saturation concentration of O_2_ (calculated based on^48^). According to^45^, the ship-based O_2_/Ar NCP estimates are highly correlated with NCP calculated from the seasonal DIC drawdown in this region (*R*^2^ = 0.83). Note that our O_2_/Ar-NCP measurements in this study only reflect the mixed layer carbon fluxes and we do not assess the sequestration timescales.

### DNA extraction and metabarcoding

DNA extraction and PCR were conducted as previously described^40^. In brief, cells were lysed by bead-beating at 4800 rpm for 1 min with 0.2 g of 0.1 mm Zr beads in 400 µl of Qiagen lysis buffer AP1. DNA was then extracted using DNeasy Plant Mini Kit (Qiagen, Valencia, CA, USA) following the manufacturer’s instructions. rRNA gene amplicon libraries were constructed using dual indexed 18S rRNA gene V4 primer set^16^, EukF (5′–CCAGCASCYGCGGTAATTCC–3′) and EukR (5′–ACTTTCGTTCTTGAT–3′). For each sample, PCR amplifications were conducted in triplicates with one blank as a control for contamination. The PCR reactions followed a 30-cycle program with annealing temperature at 57 °C. The resulting PCR products were purified using QIAquick PCR Purification Kit (Qiagen), and were pooled in equimolar concentration to 10 ng/µl approximately. The pooled amplicon libraries were sequenced at Duke Center for Genomic and Computational Biology in three MiSeq 300PE runs.

### Sequence processing

Paired-end reads with dual indices were assembled using VSEARCH v2.3.4 (Rognes et al. 2016) following the algorithm described in^49^. The merged reads were (i) demultiplexed in QIIME 1^50^; (ii) trimmed to remove Illumina adapters, primers, and barcodes, using BBDuk (v38.29) (http://jgi.doe.gov/data-and-tools/bb-tools/); and (iii) processed following the DADA2 pipeline (version 1.10.1) to infer ASVs^51^. Quality filtering and denoising with chimeras removal were performed using the incorporated functions in the DADA2 package. In total, 2480 ASVs were identified from the five-year amplicon dataset. The sequence counts per sample (after quality filtering) were reported in Supplementary Data 1, with median = 67,547 reads per sample.

ASVs were then classified by the ‘assignTaxnonomy’ function in DADA2 following the naïve Bayesian classifier method^52^, using a DADA2 formatted Silva 132 reference database (DOI 10.5281/zenodo.1172783)^53^. The resulted classification for each ASV is presented in Supplementary Data 3.

### Alpha diversity

After discarding two samples with the lowest counts 2016S33 and 1016S34, the libraries (*n* = 117) were rarified to even depth. Alpha diversity indices Chao1 and Shannon (H′) were calculated for each sample using R package Phyloseq v1.26.1^54^. Pielou’s evenness was calculated as *J* = *H*′/ln(*S*), where *S* is the total number of ASVs observed in a rarified sample.

### Canonical correspondence analysis (CCA)

CCA was conducted to investigate the relationships between community composition changes and environmental constrains^55^ using R package vegan (v2.5-4)^56^. In total, 14 environmental variables were initially examined, including XgridCal, YgridCal, PAR, Salinity, SST, Chl, mixed layer depth, volumetric NCP, biological oxygen supersaturation, PO_4_, SiO_4_, N+N, *fsim,* and *fmet*. Bacterial production (BP) and primary production (PP) were not included in this analysis due to a large number of missing values. After a stepwise variable selection based on Akaike Information Criterion (permutation = 1000 per step), the constrained community CCA was conducted with selected environmental variables and the results were presented in Fig. 3.

### Construct statistical models for NCP

Below we describe a three-step procedure: (i) ASV counts were normalized to generate QMP; (ii) in order to reduce the model dimension, a WGCNA was applied to QMP to generate modules or bio-assemblages, and (iii) the resulting WGCNA modules and environmental variables were fed to the genetic programming (GP) algorithm to construct predictive models for NCP.

#### Quantitative microbiome profiling (QMP)

All samples from the year 2014 and four samples from the year 2013 (2013SA, 2013SB, 2013SC, and 2013SD) were processed first, and 0.88 ng of *Schizosaccharomyces pombe* gDNA (ATCC #24843D-5, Manassas, VA, USA) in single-use aliquot was spiked into to each sample as an internal standard before DNA extraction. The *S. pombe* reads did not turn out high enough for normalization in the resulting library, i.e., ≤0.1%. For samples from the years 2012, 2013 (except for the previous four samples), and 2015, we increased the amount of *S. pombe* gDNA to 16.0 ng per sample and the internal standard proportion turned out appropriate for detection (0.7−5.7% of the total 18S counts). In the third batch, samples from the year 2016 were extracted with no internal standard due to a logistic issue in the lab.

For samples from the second batch, we normalized the ASV counts to QMP (in unit of 18S gene copy numbers L^−1^) using internal standards and recorded filtration volumes as described in^40^.

For samples from the first and third batches, reads were normalized to QMP using an empirical linear relationship^40^ (*R*^2^ = 0.94) between *x*—cryptophyte Alloxanthin concentrations in μg/L, and *y*—cryptophyte 18S rRNA gene counts in copies/mL: *y* = 2.05 × 10^6^*x*. The Alloxanthin concentrations in the linear calibration range from 0.01 to 6.22 μg/L. Although Alloxanthin concentrations for all samples used in this calculation are above 0.01 μg/L, we note that there is higher uncertainty towards the lower concentration end. The resulted Phaeocystis 18S QMP in years 2014 and 2016 were strongly correlated with Phaeocystis CHEMTAX abundances (*R*^2^ = 0.62), except for two outliner samples 2014S17 and 2014S44, likely due to low Alloxanthin concentrations in these two samples. QMP for these two samples was then recalculated using an empirical linear relationship derived from Phaeocystis CHEMTAX abundance^40^.

As a complementary analysis, we recalculated QMP (QMP_recal) using the empirical HPLC-CHEMTAX normalization for samples from 2013 to 2016 (no HPCL data for the year 2012). The resulted QMP_recal is highly similar to the internal standard method QMP (*y* = 0.99*x*, *R*^2^ = 0.87), except for one sample 2015S13.

#### Weighted gene correlation network analysis

ASVs which were not observed more than three times in at least 20% of the samples were removed from the count table. WGCNA was conducted to identify inter-connected plankton bio-assemblages (modules) and correlate them with environmental variables using R package WGCNA v 1.66^27^. The calculated QMP for 112 samples were included in one WGCNA run with all samples considered independent of each other. The QMP matrix was log-transformed. The detailed R codes for each step of this analysis are presented as a supplementary file. Soft thresholding power was set at 4, which was the minimum value for the scale-free topology fit reaching *R*^2^ = 0.9. In module identification using dynamic tree cut, the minimum module size was set at 10 in order to generate medium to large modules. The analysis resulted in 12 WGCNA modules from thousands of ASVs, thereby significantly reducing the number of input variables for the NCP model. The co-occurrence network of each module was visualized using an open-source tool Cytoscape (v3.7.0).

#### Genetic programming to build NCP models

Based on community structure (modules) and abiotic environmental variables, GP was used to construct statistical models predicting volumetric NCP. GP is a machine-learning approach based on evolutionary computation and it has been successfully used to construct a NCP algorithm based on satellite observations in a previous study^31^. In this study, the input factors for GP are, (i) the eigenvalues for 12 WGCNA modules, representing the biological/community factors with resolution at ASV level, and (ii) a list of physical factors, including MLD, PAR, PAR_mld, Salinity, SST, *f*_sim_, *f*_met_, X grid, and Y grid, which may directly or indirectly influence plankton physiology. The combined dataset (*n* = 112) was randomly split into even training and validation datasets. GP was then conducted using Eureqa (v1.24.0) following the recommendations by^57^. The candidate solutions with varying complexity were ranked by mean squared error  (Table S2). In order to reduce the risk of overfitting, the complexity of the candidate solutions was kept to a minimum.

### Reporting summary

Further information on research design is available in the Nature Research Reporting Summary linked to this article.

## Supplementary information

Supplementary information

Description of Additional Supplementary Files

Supplementary Data 1

Supplementary Data 2

Supplementary Data 3

Supplementary Data 4

Reporting Summary

## Data Availability

DNA sequencing data generated in this study have been deposited in the National Center for Biotechnology Information (NCBI) under accession number PRJNA508517. Palmer LTER data are available through Datazoo (http://pal.lternet.edu/data). Silva 132 reference database used for taxonomy classification was downloaded from (10.5281/zenodo.1172783). Source data are provided with this paper.

## Source data

Source Data

## Code availability

The R codes for WGCNA analysis and the MATLAB codes for NCP calculation can be accessed from GitHub (https://github.com/nicolascassar/WGCNA-Analyses and https://github.com/nicolascassar/O2Ar_calculations).
