# Supplementary material for: Decline in plankton diversity and carbon flux with reduced sea ice extent along the Western Antarctic Peninsula
Source: Nat Commun. 2021 Aug 16;12:4948. doi: 10.1038/s41467-021-25235-w (PMC8368043; doi:10.1038/s41467-021-25235-w)
Supplement: Supplementary file 1 — Supplementary information [file 41467_2021_25235_MOESM1_ESM.pdf]

Decline in plankton diversity and carbon flux with reduced sea ice extent along the Western Antarctic Peninsula

Yajuan Lin, Carly Moreno, Adrian Marchetti, Hugh Ducklow, Oscar Schofield, Erwan Delage, Michael Meredith, Zuchuan Li, Damien Eveillard, Samuel Chaffron, and Nicolas Cassar

Supplementary figures and tables;

Supplementary Figure S1: Distributions of January WAP SST and sea ice concentrations from 1982 to 2020

Supplementary Figure S2: Sea ice concentrations versus sea surface temperatures in January 1982-2020 in the WAP region

Supplementary Figure S3: Summary of community composition at the phylum level

Supplementary Figure S4: Phylum-level community composition for individual stations

Supplementary Figure S5: Relationships of WGCNA modules vs. environment parameters

Supplementary Figure S6: Module biogeography along the WAP grids from 2012 to 2016

Supplementary Table S1: Abbreviations for examined environmental variables

Supplementary Table S2: NCP solutions derived by Genetic Programming

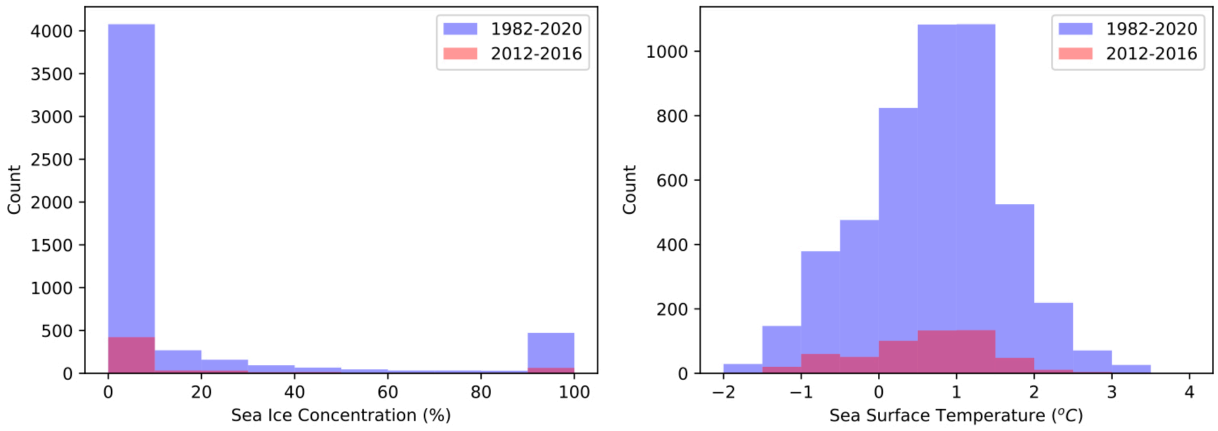

Supplementary Figure S1: Distributions of January WAP SST and sea ice concentrations from 1982 to 2020. January averaged SST (*left*) and sea ice concentrations (*right*) extracted from remote sensing data in Palmer grids. The distributions for sampled years 2012 – 2016 are shown in red. Source data are provided as a Source Data file.

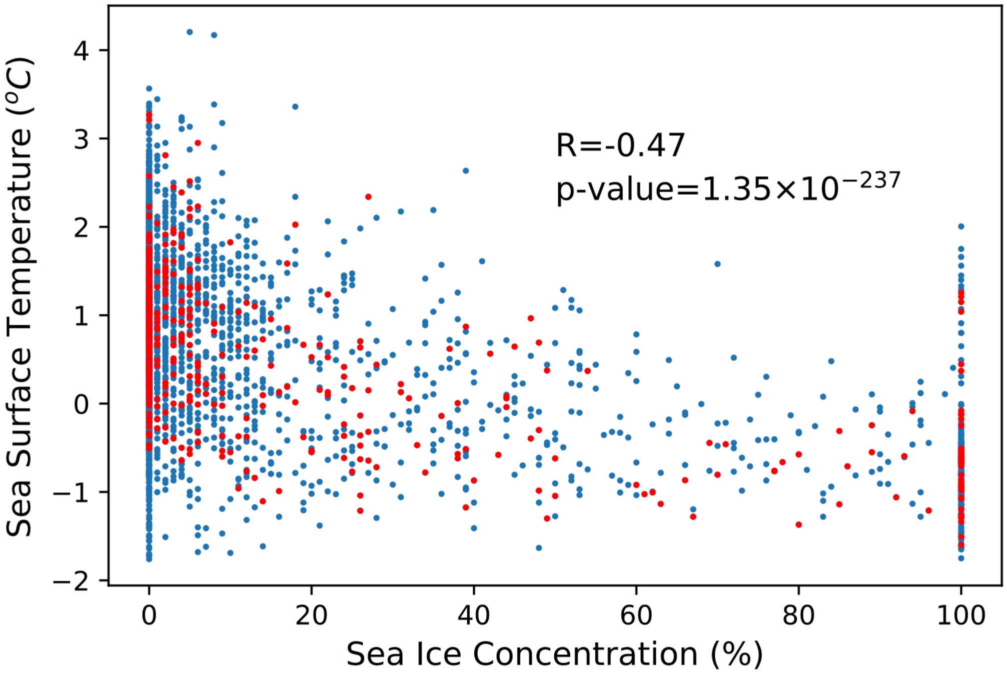

Supplementary Figure S2: Sea ice concentrations versus sea surface temperatures in January 1982-2020 in the WAP region. Shown in text are Pearson correlation coefficient and p-value (two-sided t-test) for the whole dataset 1982-2020. Values for data in sampled years 2012-2016 are shown in red. Source data are provided as a Source Data file.

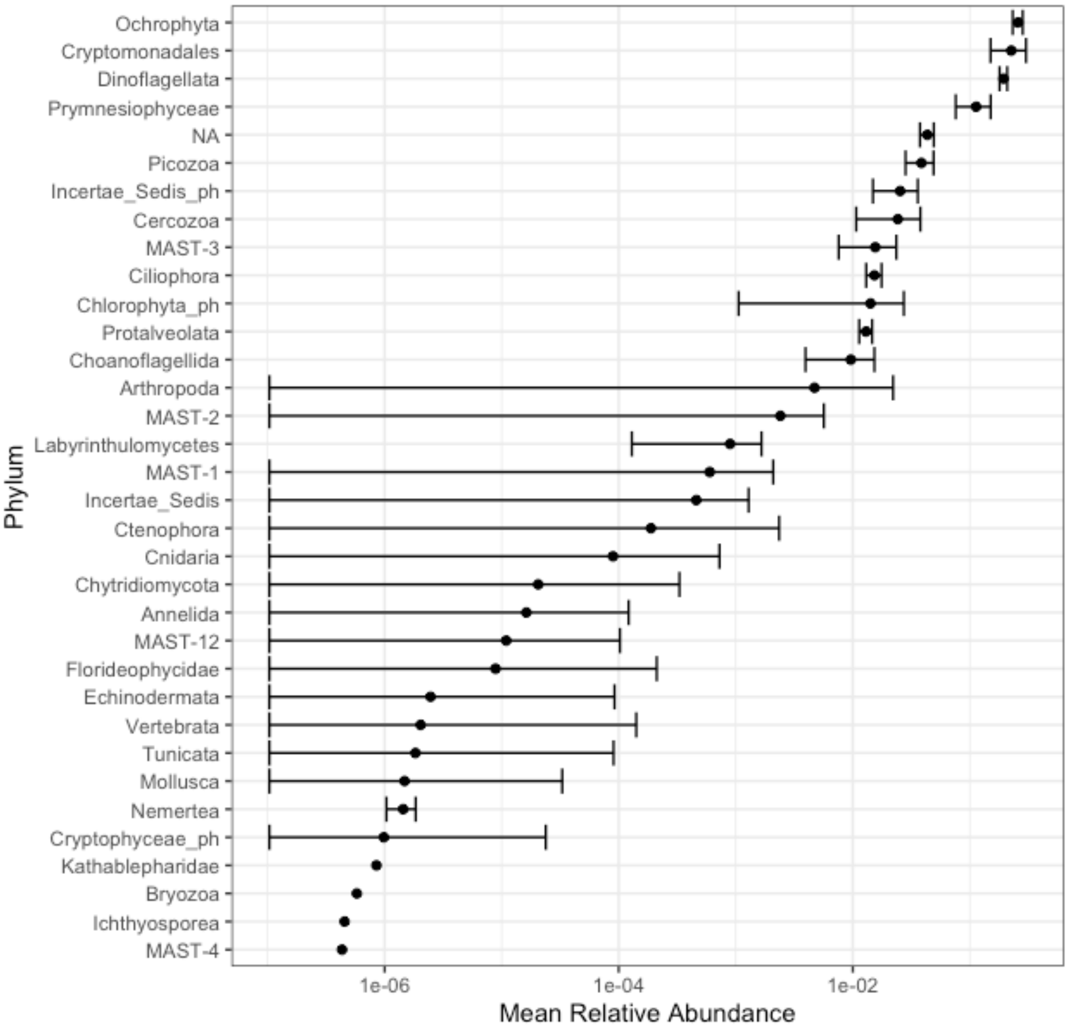

Supplementary Figure S3: Summary of community composition at the phylum level. Relative abundances of different taxa were inferred from 18S rRNA gene sequences using DADA2 curated Silva v132 database. The total sample n = 119, including n = 23 for year 2012, 23 for year 2013, 25 for year 2014, 18 for year 2015 and 30 for year 2016. Relative abundances for each phylum are presented as mean values with error bars representing standard deviations. Ochrophyta, the most abundant phylum for these WAP samples, mainly consist of diatoms (87% in 18S rRNA copies). Source data are provided as a Source Data file.

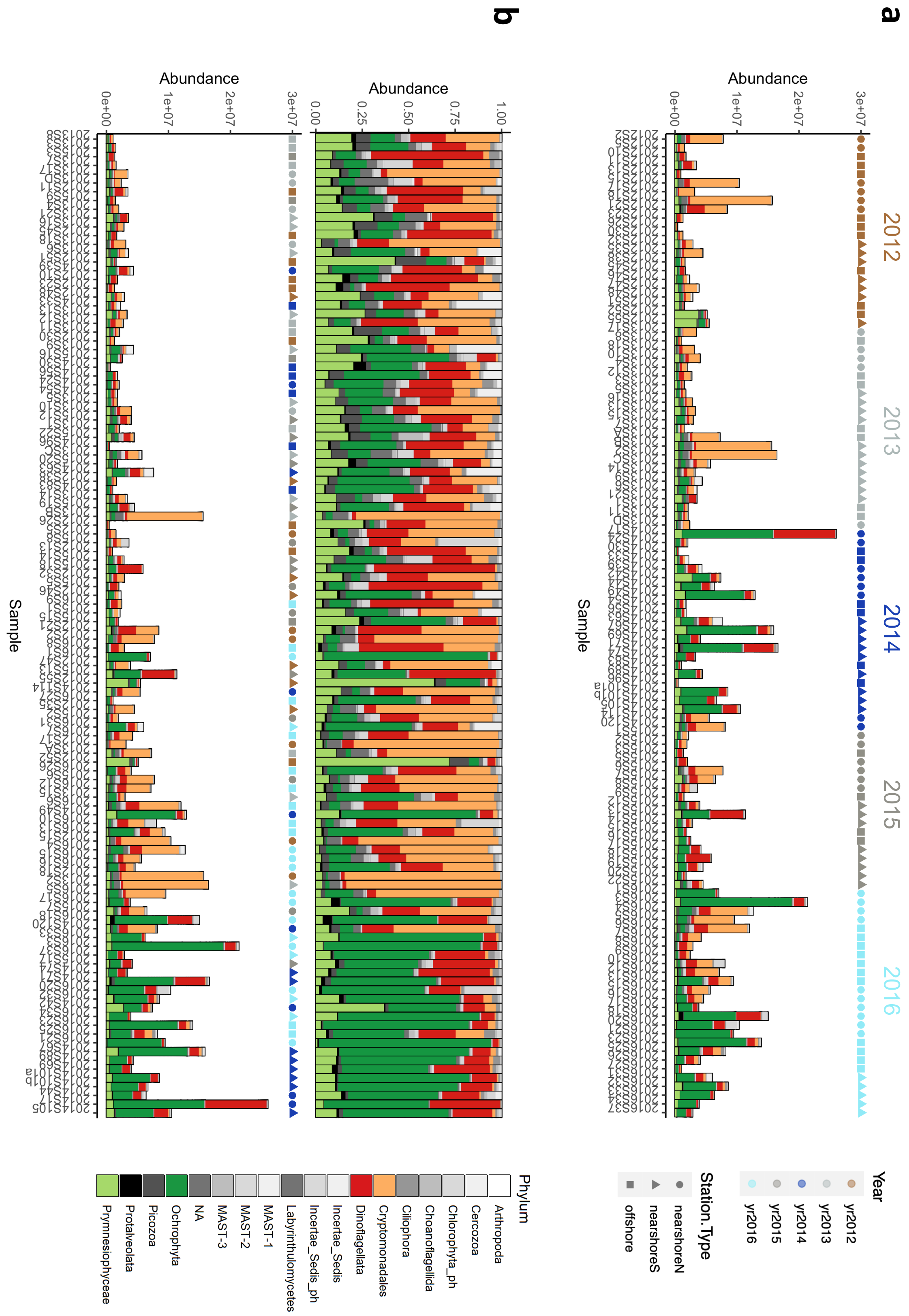
a
b
2012
2013
2014
2015
2016
Abundance
Sample
0e+00
1e+07
2e+07
3e+07
0.00
0.25
0.50
0.75
1.00
Year
yr2012
yr2013
yr2014
yr2015
yr2016
Station.Type
nearshoreN
nearshoreS
offshore
Phylum
Arthropoda
Cercozoa
Chlorophyta_ph
Choanoflagellida
Ciliophora
Cryptomonadales
Dinoflagellata
Incertae_Sedis
Incertae_Sedis_ph
Labyrinthulomycetes
MAST-1
MAST-2
MAST-3
NA
Ochrophyta
Picozoa
Protalveolata
Prymnesiophyceae

Supplementary Figure S4: Phylum-level community composition for individual stations. Five-year community compositions at the WAP are presented as bar plots, with points on top of the bars indicating sampling year by color and station type by shape. **a.** Stations ranked by sampling time. In particular, samples are separated into warm years (2012, 2013 and 2015) and cold years (2014 and 2016). **b.** Stations ranked in ascending Chlorophyll (Chl), as an index for biomass. In addition to the traditional relative abundance profiling (Panel **b**, *upper*), we conducted quantitative microbiome profiling (QMP) using an internal DNA standard method. Calculated QMP in 18S copies ml^{-1} is shown in Panel **a** and Panel **b,** *lower*.

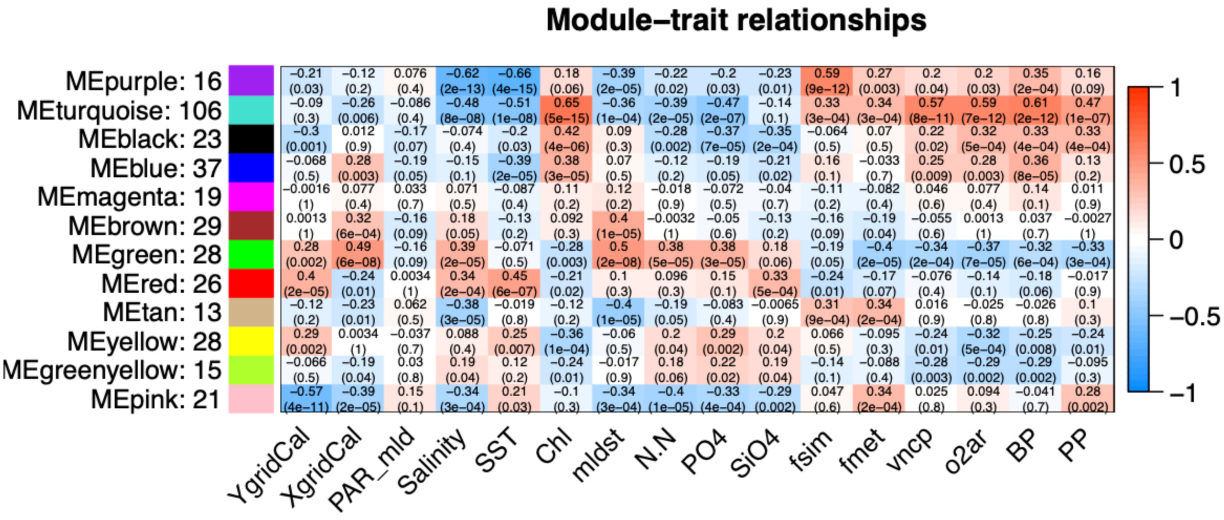

Supplementary Figure S5: Relationships of WGCNA modules vs. environment parameters. Using WGCNA algorithm, communities were decomposed into 12 subnetworks/modules coded by different colors showing on the y-axis. Numbers next to the module names represent the number of ASVs included in each module. Each row in the matrix corresponds to a module, and each column to an environmental variable. The correlation coefficients are represented by colors according to the color bar as well as text in each grid with p-values (two-sided t-test) in parentheses. Acronyms for a list of examined environmental factors are listed in Table S1.

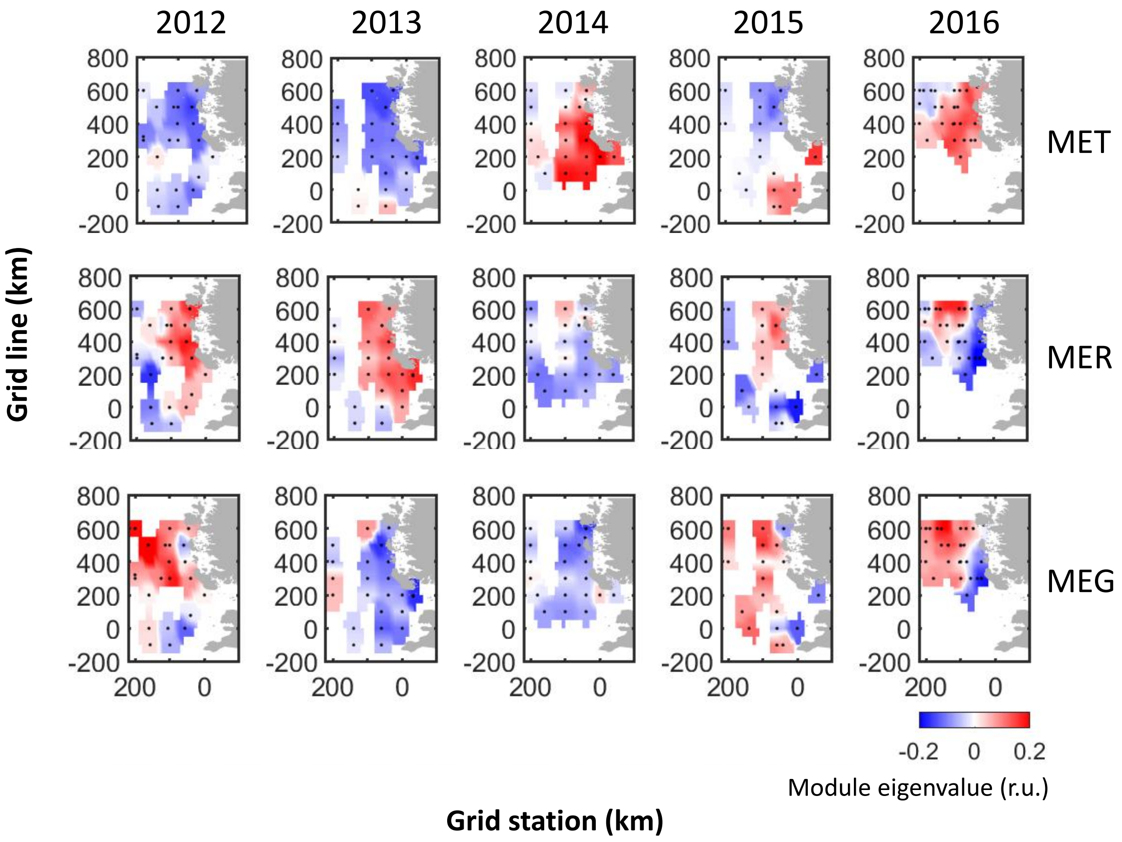

Supplementary Figure S6: Module biogeography along the WAP grids from 2012 to 2016. The biogeography of three modules MET (*top*), MER (*middle*) and MEG (*bottom*), with color indicating the module eigenvalues in relative unit. Source data are provided in Supplementary Data 4.

Supplementary Table S1: Abbreviations for examined environmental variables

Acronyms	Environmental variables	Units
SST	Sea surface temperature	ºC
PAR	Photosynthetically active radiation	μmol (photons) m^{-2} s^{-1}
PAR_mld	Mixed layer depth averaged photosynthetically active radiation	μmol (photons) m^{-2} s^{-1}
PO_4	Phosphate concentrations	μM
N.N	Nitrate plus nitrite concentrations	μM
SiO_4	Silicate concentrations	μM
mldst	Mixed layer depth defined by potential density (sigma-theta)	m
Salinity	Salinity	PSU
XgridCal	X grid or grid station calculated from GPS	km
YgridCal	Y grid or grid line calculated from GPS	km
Chl	Underway chlorophyll concentrations estimated by fluorometer	μg L^{-1}
fsim	Fraction of sea-ice melt water ($\delta^{18}O$)	%
fmet	Fraction of meteroic water ($\delta^{18}O$)	%
o2ar	Underway biological oxygen supersaturation	%
vncp	Underway volumetric net community production	mmol O_2 m^{-3} d^{-1}
BP	Bacterial production (leucine incorporation)	pmol L^{-1} $hour^{-1}$
PP	Primary production	mg m^{-3} day^{-1}

Supplementary Table S2: NCP solutions derived by Genetic Programming

Solutions	R^2	MSE
$\frac{7.02+41.1\,MET}{0.361mld-1.13-1.08SST}-0.14$ (1)	0.70	1.18
$1.91MER+\frac{7.2+45.5\,MET}{0.366mld-1.13-1.08SST}-0.144$ (2)	0.72	1.12
$0.255+\frac{3.55+42.2\,MET+2.06SST+0.0029PAR}{14.8MEG+0.575mld-3.78-0.0257\,PAR\,MEG}-0.14$ (3)	0.79	0.85
$0.209+1.91MER+\frac{4.14+46.1MET+2.06SST+0.0029PAR}{15.6MEG+0.579mld-3.6-0.0271\,PAR\,MEG}-1.91MEGY$ (4)	0.80	0.79
