# Supplementary material for: Decline in plankton diversity and carbon flux with reduced sea ice extent along the Western Antarctic Peninsula
Source: Nat Commun. 2021 Aug 16;12:4948. doi: 10.1038/s41467-021-25235-w (PMC8368043; doi:10.1038/s41467-021-25235-w)
Supplement: Supplementary file 2 — Description of Additional Supplementary Files [file 41467_2021_25235_MOESM2_ESM.docx]

**Description of Additional Supplementary Files**

Title: Supplementary Data 1

Description: Sample metadata and read counts

Title: Supplementary Data 2

Description: List of WGCNA module memberships and significance for NCP

Title: Supplementary Data 3

Description: Taxonomic classification

Title: Supplementary Data 4

Description: Module eigen values from WGCNA
