# Supplementary material for: Decline in plankton diversity and carbon flux with reduced sea ice extent along the Western Antarctic Peninsula
Source: Nat Commun. 2021 Aug 16;12:4948. doi: 10.1038/s41467-021-25235-w (PMC8368043; doi:10.1038/s41467-021-25235-w)
Supplement: Supplementary file 7 — Reporting Summary [file 41467_2021_25235_MOESM7_ESM.pdf]

nature research

Corresponding author(s): Yajuan Lin; Nicolas Cassar

Last updated by author(s): Jul 17, 2021

Reporting Summary

Nature Research wishes to improve the reproducibility of the work that we publish. This form provides structure for consistency and transparency in reporting. For further information on Nature Research policies, see our Editorial Policies and the Editorial Policy Checklist.

Statistics

For all statistical analyses, confirm that the following items are present in the figure legend, table legend, main text, or Methods section.

n/a	Confirmed	
☐	☒	The exact sample size (*n*) for each experimental group/condition, given as a discrete number and unit of measurement
☐	☒	A statement on whether measurements were taken from distinct samples or whether the same sample was measured repeatedly
☐	☒	The statistical test(s) used AND whether they are one- or two-sided<br>*Only common tests should be described solely by name; describe more complex techniques in the Methods section.*
☐	☒	A description of all covariates tested
☐	☒	A description of any assumptions or corrections, such as tests of normality and adjustment for multiple comparisons
☐	☒	A full description of the statistical parameters including central tendency (e.g. means) or other basic estimates (e.g. regression coefficient) AND variation (e.g. standard deviation) or associated estimates of uncertainty (e.g. confidence intervals)
☐	☒	For null hypothesis testing, the test statistic (e.g. *F*, *t*, *r*) with confidence intervals, effect sizes, degrees of freedom and *P* value noted<br>*Give P values as exact values whenever suitable.*
☒	☐	For Bayesian analysis, information on the choice of priors and Markov chain Monte Carlo settings
☒	☐	For hierarchical and complex designs, identification of the appropriate level for tests and full reporting of outcomes
☐	☒	Estimates of effect sizes (e.g. Cohen's *d*, Pearson's *r*), indicating how they were calculated

Our web collection on statistics for biologists contains articles on many of the points above.

Software and code

Policy information about availability of computer code

Data collection	Silva 132 reference database used for taxonomy classification was downloaded from (DOI 10.5281/zenodo.1172783). NCP code is available from GitHub (https://github.com/nicolascassar/O2Ar_calculations).
Data analysis	MATLAB (R2018b and R2020a), Microsoft Excel (2019), R package DADA2 (v. 1.10.1), R package Phyloseq (v1.26.1), R package Vegan (v 2.5-4), R package WGCNA (v1.66), VSEARCH (v2.3.4), QIIME 1 (v1.7.1), BBDuk(v38.29), Eureqa-Formulize (v1.24.0), Cytoscape (v3.7.0), Customized MATLAB codes for NCP available from GitHub (https://github.com/nicolascassar/O2Ar_calculations)

For manuscripts utilizing custom algorithms or software that are central to the research but not yet described in published literature, software must be made available to editors and reviewers. We strongly encourage code deposition in a community repository (e.g. GitHub). See the Nature Research guidelines for submitting code & software for further information.

Data

Policy information about availability of data

All manuscripts must include a data availability statement. This statement should provide the following information, where applicable:

- Accession codes, unique identifiers, or web links for publicly available datasets
- A list of figures that have associated raw data
- A description of any restrictions on data availability

DNA sequencing data generated in this study have been deposited in the National Center for Biotechnology Information (NCBI) under accession number PRJNA508517. Palmer LTER data are available through Datazoo (http://pal.lternet.edu/data). Silva 132 reference database used for taxonomy classification was downloaded from (DOI 10.5281/zenodo.1172783).

Field-specific reporting

Please select the one below that is the best fit for your research. If you are not sure, read the appropriate sections before making your selection.

☐ Life sciences ☐ Behavioural & social sciences ☒ Ecological, evolutionary & environmental sciences

For a reference copy of the document with all sections, see nature.com/documents/nr-reporting-summary-flat.pdf

Ecological, evolutionary & environmental sciences study design

All studies must disclose on these points even when the disclosure is negative.

Study description	Using plankton DNA samples collected from the PAL LTER annual cruise during 2012-2016 to understand the biodiversity and carbon flux change associated with environmental change
Research sample	DNA samples were collected from surface sea water along the PAL LTER annual cruise tracks and sequenced to exam eukaryotic plankton community composition and diversity.
Sampling strategy	Discrete DNA samples were taken from sea water following the LTER sampling grids during the annual cruises. On average 23 samples were processed and sequenced each year covering most planned PAL LTER cruise CTD stations. This selection is to match DNA sample with the CTD oceanography dataset. In addition, for WGCNA analysis, the minimum sample size is suggested to be 20.
Data collection	Yajuan Lin, Carly Moreno, Hugh Ducklow, Michael Meredith and Oscar Schofield collected the field samples and underway data. The underway O2/Ar data were measured by an equilibrator inlet mass spectrometer (EIMS) recorded in real time and saved as Excel files for later analysis in MATLAB.
Timing and spatial scale	Sampling time: Jan-Feb each year during 2012-2016<br>Spatial scale: PAL LTER regional sampling grids west of the Antarctic Peninsula<br>The sampling time period is designed to match the most productive time of the year at the WAP. The PAL LTER grids were designed to cover large environmental/ecological gradients from North to South and from coast shelf zone to open ocean at the WAP.
Data exclusions	Two samples with the lowest sequencing counts (<8k per sample) were excluded from the diversity analysis
Reproducibility	Experimental replication is not applicable to this study because we conduct environmental sampling/measurements
Randomization	We included all environmental samples as independent biological samples for analyses. No groups or controls were defined
Blinding	Data acquisition and analyses were blind

Did the study involve field work? ☒ Yes ☐ No

Field work, collection and transport

Field conditions	Coastal Antarctica region in austral summer, sea surface temperature between -2 to 3 degree C
Location	80W - 55W, 62S - 70S; surface seawater from depth about 5m
Access & import/export	Sampling efforts and import/export logistics were supported by the United States Antarctic Program and the Office of Export Controls at Duke University
Disturbance	Potential disturbance to the marine environment: research vessel gas emissions and noises. Operations only conducted when necessary for proposed research activities; energy efficiency and water usage efficiency on board; no solid waste or chemical disposal allowed on board

Reporting for specific materials, systems and methods

We require information from authors about some types of materials, experimental systems and methods used in many studies. Here, indicate whether each material, system or method listed is relevant to your study. If you are not sure if a list item applies to your research, read the appropriate section before selecting a response.

Materials & experimental systems

n/a	Involved in the study
☒	☐ Antibodies
☒	☐ Eukaryotic cell lines
☒	☐ Palaeontology and archaeology
☒	☐ Animals and other organisms
☒	☐ Human research participants
☒	☐ Clinical data
☒	☐ Dual use research of concern

Methods

n/a	Involved in the study
☒	☐ ChIP-seq
☒	☐ Flow cytometry
☒	☐ MRI-based neuroimaging
